# Comparison of Laparoscopic Ovarian Drilling Success between Two Standard and Dose-Adjusted Methods in Polycystic Ovary Syndrome: A Randomized Clinical Trial

**DOI:** 10.22074/ijfs.2020.5628

**Published:** 2019-11-11

**Authors:** Leili Hafizi, Maliheh Amirian, Yasmin Davoudi, Mona Jaafari, Ghazal Ghasemi

**Affiliations:** 1 Department of Obstetrics and Gynaecology, Faculty of Medicine, Mashhad University of Medical Sciences, Mashhad, Iran; 2 Department of IVF and Infertility, Faculty of Medicine, Mashhad University of Medical Sciences, Mashhad, Iran; 3 Department of Radiology, Faculty of Medicine, Mashhad University of Medical Sciences, Mashhad, Iran

**Keywords:** Anti-Müllerian Hormone, Infertility, Polycystic Ovary Syndrome

## Abstract

**Background:**

One of the treatment methods for increasing the ovarian response to ovulation induction in polycystic ovary syndrome (PCOS) is laparoscopic ovarian drilling (LOD). The optimal amount of the electrosurgical energy discharged in the ovaries to achieve maximum treatment response with minimal follicle injury is unknown. This study was performed to compare the success level of LOD by means of standard and dose-adjusted treatment methods among infertile clomiphene-resistant PCOS women.

**Materials and Methods:**

This randomized clinical trial was conducted on infertile clomiphene citrate-resistant PCOS women in the Gynaecology Department of Imam Reza Hospital between 2016 and 2017. The patients were randomly di- vided into two groups based on the ovarian cautery method. The two groups were examined and compared regarding the antral follicles, the serum levels of anti-Müllerian hormone (AMH), androgens, and mid-luteal progesterone one month after surgery. The regularity of cycles, ovulation, and pregnancy were examined monthly up to six months after surgery.

**Results:**

In total, 60 women received bilateral LOD (n=30 per group). The level of AMH (P=0.73), testosterone (P=0.91), and dehydroepiandrosterone sulphate (DHEAS, P=0.16) did not differ at study entrance and one month after ovarian cautery [P=0.94 (AMH), P=0.46 (testosterone), and P=0.12 (DHEAS)] and for postoperative mid-luteal pro- gesterone (P=0.31). Intragroup comparisons showed a statistically significant difference in the decrease in the number of antral follicles and testosterone in the standard group (P=0.02) and AMH level in the cautionary dose-adjusted group (P=0.04). We observed no difference in cycle regularity (P=0.22), ovulation (P=0.11), and pregnancy (P=0.40) between the two groups after six months.

**Conclusion:**

The results indicated that there was no difference between the two methods of ovarian cautery with re- gards to establishing cycle regularity and ovulation. The standard treatment was effective in decreasing the numbers of antral follicles and testosterone levels, whereas the dose-adjusted method significantly affected the decrease in AMH levels (Registration Number: IRTC20171210037820N1).

## Introduction

Polycystic ovary syndrome (PCOS) was initially reported by Stein and Leventhal (1) in modern medical texts when they described seven women who suffered from amenorrhea, hirsutism, and enlarged ovaries that contained several cysts. This syndrome is now considered to be a common, heterogeneous, and hereditary disorder which can affect women of reproductive age. The prevalence of PCOS may vary based on the applied diagnostic criteria (2, 3). The highest rate of PCOS has been reported at 52% among West Asian women having migrated to England (4); in other references, this figure has been reported as 2-26% (3). Infertility involves 40% of the cases affected by PCOS (5).

Clomiphene citrate is the first treatment option for inducing ovulation in these women (6-8); however, drug resistance has been observed in around 20% of such cases (8). Clomiphene resistance is defined as three cycles of ovulation failure or six cycles without pregnancy (9). One of the alternatives used among clomiphene-resistant women is laparoscopic ovarian drilling (LOD); particularly, in cases which the patient has other surgical indications or when she is unable to attend the frequent visits required for treatment with gonadotropins (10).

Historically, the surgical treatment of infertile PCOS women reported by Stein and Leventhal (1) in 1935 was ovarian wedge resection via laparotomy, and it showed promising results. However, three decades later, this method was abandoned due to the risk of pelvic adhesions following surgery and has been replaced by ovulation-inducing medications such as clomiphene and gonadotropins (11). In 1984, the surgical treatment of infertile PCOS women improved remarkably with the introduction of LOD that had an ovulation success rate of 92% and pregnancy success rate of 80% (12). LOD, as a less harmful and less-invasive method compared to ovarian wedge resection, uses electrocautery (diathermy) or laser beam and has played a significant role in the treatment of infertile PCOS women (11).

The beneficial effects of this method seem to be related to the destruction of the androgen generating stroma, which results in reduced production of androgens in the ovary and its reduced concentration in the blood circulation. Clomiphene citrate-resistant women may respond better to medical therapy after this type of surgery. Sensitivity to exogenic gonadotropins also increase in such cases (13).

Several studies have evaluated ovarian cauterization. In the initial studies, it was hypothesized that a higher energy level would result in a more efficient procedure. Subsequently, lower temperatures with a fixed number of drilled points, regardless of the ovary’s size or unilateral ovarian cautery have been reported with the intent to reduce a possible risk of ovarian atrophy and adnexal adhesions. With such fixed doses of temperature, the optimal amount of ovulation may not be achieved or the clinical manifestations of the disease may persist in individuals with enlarged ovaries (14). Armar et al. (15) reported the first descriptive research on ovarian drilling with 4 drills at a dose of 640 joules per ovary; this method was later widely accepted and used in various studies. Many authors subsequently examined and compared the effects and consequences of changes in the number of ovarian drills or the appropriate thermal dose based on the ovarian size during laparoscopic ovarian cauterization (14-22). However, in some studies, the relationship between the number of ovarian drills and adnexal adhesion was not confirmed (23). Nevertheless, the optimal amount of electrosurgical energy required during LOD to achieve the maximal fertility outcome without causing any risk to the follicles and ovaries has not been established (24).

We designed this study because of the inadequate number of studies in this area (particularly in Iran) and by taking into consideration the influence of genetic, regional and nutritional factors on PCOS. We sought to compare the effect of ovarian cauterization between the standard and dose-adjusted (based on the ovarian volume) methods in Iranian women with infertile clomiphene-resistant PCOS.

## Materials and Methods

This randomized clinical trial was conducted in the Gynaecology Department of Imam Reza Hospital, Mashhad, Iran from 2016 to 2017. All infertile clomipheneresistant PCOS women who visited the Gynaecology Department enrolled in this study. The sample size of this study was calculated at 30 women according to the following formula and by taking into consideration information from a previously published study (14), with an alfa error=0.05, beta error=0.8, P1=0.6, and P2=0.9. 

n=(Z1-α2+Z1-β)2(P1(1-P1)+P2(1-P2))(P1-P2)2

The achieved power of this study was 37% based on the antral follicle count (AFC).

## Ethical observations 

At study initiation, the study protocol was fully described to each patient and they were free to withdraw from the study at any time. Their data was regarded as confidential. All patients signed a written informed consent to participate in the study. The Ethics Committee of Mashhad University of Medical Sciences approved this study (IR.MUMS.fm.REC.1395.335). The study was registered in the Iranian Registry for Clinical Trials (IRCT20171210037820N1).

The inclusion criteria were: all women aged 18 to 35 years, not pregnant despite two years without contraception, diagnosed with PCOS based on the Rotterdam criteria, having ruled out other reasons of infertility except for ovulation disorder (normal sperm analysis of the spouse, normal uterine tubes in hysterosalpingography or laparoscopy), clomiphene-resistant, and provided consent to participate in this study.

Exclusion criteria were: withdrawal during the study, patients lost to follow-up, presence of any other pathology during laparoscopy (e.g., endometriosis or adhesion) suggestive of other aetiologies for infertility.

Initially, we recorded the patients’ demographic characteristics and paraclinical data by means of an interview and the patient’s records. We divided the patients into two groups according to a table of random number generator with equal sizes of groups: standard method (group A) and ovarian cautery based on the ovarian volume or the dose-adjusted method (group B). One radiologist performed the transvaginal ultrasonography (TVS) for group B patients by using a Honda sonography device (Honda Electronics, Japan) to measure ovarian volume. This volume was measured on the basis of a cubic centimetre and at three perpendiculars. 

A gynaecology laparoscopist performed each laparoscopy via an Olympus laparoscopic machine (Olympus Europa SE & Co., Germany) in the gynaecology theatre of Imam Reza Hospital with patients under general anaesthesia and in the lithotomy position. Abdominal entry was done by the closed technique and via a Veress needle. Only patients who had any history of abdominal surgery had an open laparoscopic procedure. A triple puncture laparoscopy was performed with 3 trocars. The abdominal and pelvic environment, and the patency of the tubes were examined. Patients with adhesions, endometriosis, or any pathology in the pelvic area were excluded from the study.

Next, the ovarian cautery was performed. The uteroovarian ligament was caught with an Atraumatic Grasper (Aesculape Inc., USA) and the ovary was separated from the intestines. Afterwards, the ovarian cautery was carried out with a 4-millimetre monopolar needle electrode (with a straight needle) and with a Vallylab generator that had a voltage of 30 (in both groups) as follows: using the CUT energy, a puncture with the depth of 4 millimetres was initially created on the ovarian capsule and then the coagulation button was activated. After the cautery of each ovary and before releasing the utero-ovarian ligament, the ovary was rinsed with cold normal saline serum to prevent any adhesion or injury to the adjacent viscera. The ligament was then released and examined with regards to the possibility of mechanical injury.

In group B (on the basis of ovarian volume), the measurement of energy was based on the following model (15, 16, 18, 25) that used 640, 450, 600, and 800 joules for each ovary (mean: 625 joules) and ovarian volume means of 8 and 10 cm^3^ . The dose of 60 joules was chosen for each cubic centimetre of the ovarian mass. The ovarian mass was multiplied by 60 joules. In order to achieve the correct time, we multiplied the ovarian volume by 2 and measured the number and time of each puncture as follows: Energy=Power (voltage)×Time (number of punctures×time of each puncture) and by taking into account that the generator’s energy for all individuals was 30 joules.

In group A, based on the size of the ovary, we created either 4 drills of 5 s or 5 drills of 4 s with a voltage of 30 in order to achieve an energy of 600 joules per ovary (4×5×30=600).

The patients were followed for six months from the first menstrual cycle after the operation. Hormonal levels of anti-Müllerian hormone (AMH), testosterone, dehydroepiandrosterone sulphate (DHEAS), and progesterone were obtained on the third day of the first menstrual cycle after the operation and the progesterone level was measured at the mid-luteal phase of the same cycle. All tests were performed in the same laboratory and without charge.

TVS was also performed from the sixth day of the first menstrual cycle after the operation, every three days up to the 16^th^ day or until the observation of a dominant follicle. Ovulation was confirmed by the observation of an 18 mm dominant follicle or pregnancy. TVS was performed by the same radiologist as before the LOD. In the event of anovulation, subsequent sonographies were not performed. Cycle regularity and the occurrence of pregnancy were examined after six months.

Patients who did not menstruate until a month after ovarian cautery (menstrual cycle over 35 days) were administered 100 mg of intramuscular micronized progesterone to re-establish the menstrual cycle, and the investigations were performed though most of these cases had no ovulation.

## Statistical analysis

We performed SPSS software (version 16) analyses of te raw data. If the quantitative variables were normally distributed, we used the t test and paired t test; for non-normally distributed data, the corresponding non-parametrical tests were used. The chi-square test was used to examine qualitative variables. Repeated measure ANOVA was performed to assess the interaction and overall effect of before/ after assessments in the two groups. The significance level was set at P<0.05.

## Results

**Fig 1:**
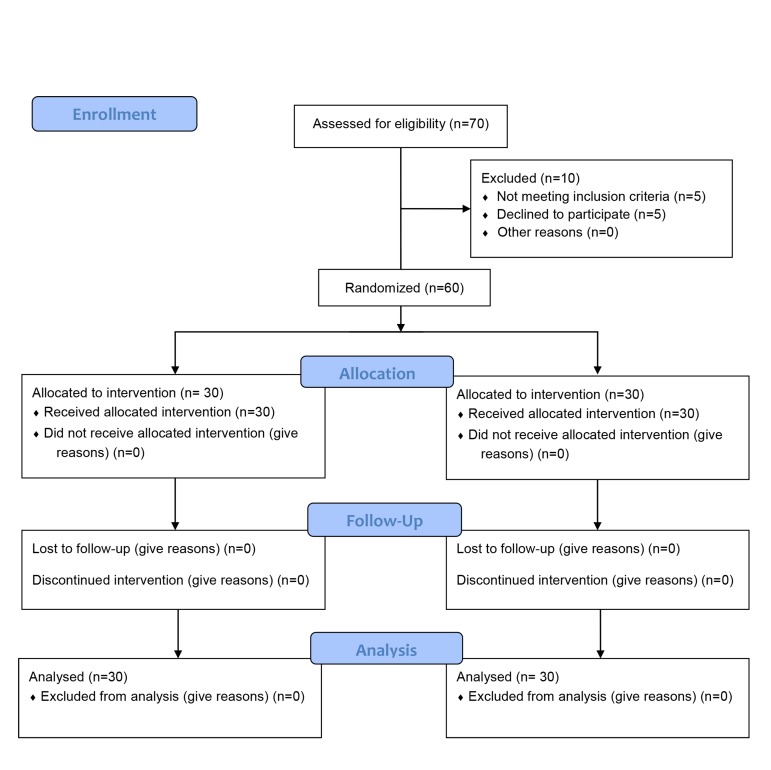
Flowchart.

In this study, 60 infertile clomiphene-resistant PCOS women received LOD by two methods: standard and dose-adjusted on the basis of the ovarian volume. The demographic, clinical and sonographic characteristics did not differ between the two study groups (Table 1, Fig.1

**Table 1:** Comparison of demographic, clinical, and sonographic data of infertile clomiphene-resistant PCOS women between the two treatment groups


Variable		Standardn=30	Dose-adjusted n=30	P value

Age (Y)		26.36 ± 4.69	28.53 ± 5.84	0.11
History of infertility (Y)		4.42 ± 2.77	4.84 ± 2.73	0.62
Type of infertility				0.79
	Primary	17 (56.6)	18 (60)	
	Secondary	13 (43.4)	12 (40)	
Clinical manifestations		20 (66.7)	19 (63.3)	0.58
	Oligomenorrhea	1 (3.3)	3 (10)	
	Oligomenorrhea+Hirsutism Hirsutism	9 (30)	8 (26.7)	
Regularity of cycles				0.61
	Regular	9 (30)	8 (26.7)	
	Irregular	21 (70)	22 (73.3)	
Sonography findings				--
	Volume of right ovary (cm^3^)	--	15.02 ± 7.46	
	Volume of left ovary( cm^3^)	--	13.34 ± 5.87	
	Endometrial line (mm)	--	6.56 ± 1.93	
AFC		16.33 ± 2.53	16.80 ± 1.99	0.43
Hormonal profile				
	AMH (ng/ml)	7.87 ± 4.86	7.46 ± 4.45	0.73
	Testosterone (ng/dl)	80.52 ± 40.80	81.46 ± 29.14	0.91
	DHEAS (µg/dL)	173.86 ± 73.32	201.34 ± 77.76	0.16


Data represented as mean ± SD or frequency (%) as appropriate. PCOS; Polycystic ovary syndrome, AMH; Anti-Müllerian hormone, AFC; Antral follicle count, and DHEAS; Dehydroepiandrosterone sulphate.

**Table 2:** Mean numbers of points and cautery time per ovary in the dose-dependent group


Variable	Right ovary	Left ovary

No. of points	5.93 ± 1.66	5.59 ± 1.68
Time (seconds)	4.07 ± 0.45	4.24 ± 0.57


Data are presented as mean ± SD.

Table 3 shows a comparison of the mean AFC and serum levels of AMH, testosterone, and DHEAS between the two groups, before and after the operation.

Table 4 displays the mean changes in the AFC and serum levels of AMH, testosterone, and DHEAS before and after the operation in each of the studied groups. Repeated measure ANOVA revealed that there was no interaction, nor any difference between the two study groups in terms of AFC (P=0.14), AMH (P=0.71), testosterone (P=0.67), and DHEAS (P=0.12).

The number of antral follicles before the operation was not significantly different between the two groups (independent t test, P=0.43). The same result was obtained after the operation (P=0.10). Intra-group comparisons showed that the decrease in the number of antral follicles was significant in the standard treatment group (paired t test, P=0.02); however, we did not observe any difference in the dose-adjusted group (P=0.24).

Before the intervention, the two groups were matched in terms of AMH level (independent t test, P=0.73). We observed the same result after the intervention (Mann-Whitney test, P=0.94). In the intra-group comparison, there was a significant decrease in the AMH level in the dose-adjusted group (paired t test, P=0.04); however, this difference was not observed in the standard treatment group (paired t test, P=0.17).

Before the intervention, the testosterone level was similar in the two groups (independent t test, P=0.91). The same result was obtained after the intervention (P=0.46). However, the decrease in testosterone level in the standard treatment group was meaningful (paired t test, P=0.02), but this difference was not observed in the dose-adjusted group (paired t test, P=0.14).

**Table 3:** Comparison of the hormone profile and AFC between the study groups


Variable	Before surgery			After surgery		
	Standard n=30	Dose-adjusted n=30	P value	Standard n=30	Dose-adjusted n=30	P value

AFC	16.33 ± 2.53	16.80 ± 1.99	0.43	15.10 ± 2.97	16.27 ± 2.55	0.10
AMH (ng/ml)	7.87 ± 4.86	7.46 ± 4.45	0.73	7.08 ± 4.28	6.71 ± 3.32	0.94
Testosterone (ng/dl)	80.52 ± 40.80	81.46 ± 29.14	0.91	71.28 ± 36.17	77.37 ± 26.68	0.46
DHEAS (µg/dL)	173.86 ± 73.32	201.34 ± 77.76	0.16	160.51 ± 60.36	189.13 ± 80.33	0.12


AFC; Antral follicle count, AMH; Anti-Müllerian hormone, and DHEAS; Dehydroepiandrosterone sulphate. Data represented as mean ± SD.

**Table 4:** A comparison of the changes in the hormone profile and AFC before and after the operation in the two groups


Variable	Standard			Dose-adjusted		
	Before surgery	After surgery	P value	Before surgery	After surgery	P value

AFC	16.33 ± 2.53	15.10 ± 2.97	0.02	16.80 ± 1.99	16.27 ± 2.55	0.24
AMH (ng/ml)	7.87 ± 4.86	7.08 ± 4.28	0.17	7.46 ± 4.45	6.71 ± 3.32	0.04
Testosterone (ng/dl)	80.52 ± 40.80	71.28 ± 36.17	0.02	81.46 ± 29.14	77.37 ± 26.68	0.14
DHEAS (µg/dL)	173.86 ± 73.32	160.51 ± 60.36	0.16	201.34 ± 77.76	189.13 ± 80.33	0.08


AFC; Antral follicle count, AMH; Anti-Müllerian hormone, and DHEAS; Dehydroepiandrosterone sulphate. Data represented as mean ± SD.

In addition, both at study initiation and study termination, the level of DHEAS did not significantly differ between the two studied groups (independent t test, P=0.16 at study initiation, P=0.12 at study termination). In the intragroup comparisons, the level of DHEAS decrease was not significant in either group (paired t test, P=0.16 in the standard group and P=0.08 in the dose-adjusted group).

The status of cycle regularity and the occurrence of ovulation and pregnancy among patients were examined from the first post-surgical cycle up to six months. The obtained results are presented in Table 5.

There were regular menstrual cycles reported in 25 (83.3%) patients in the standard treatment group and 21 (70%) patients in the dose-adjusted group. Accordingly, there was no statistically significant difference observed between the two groups (chi-square test, P=0.22).

Ovulation occurred in 26 (86.7%) patients in the standard group and in 21 (70%) patients from the doseadjusted group, which was not statistically significant (chi-square test, P=0.11).

Finally, 11 (36.7%) patients in the standard group and 8 (26.7%) patients in the dose-adjusted group became pregnant during 6 months, which indicated no meaningful difference between the two groups (chi-square test, P=0.40).

Intragroup comparisons on cycle regularity indicated a significant increase after the operation compared to before the operation in the standard treatment (30 vs. 83.3%) and dose-adjusted (26.7 vs. 70%, P<0.001) groups.

No case of premature ovarian failure was observed in our study population. Cycle regularity was experienced in 9 (30%) patients in the standard group before the operation and in 25 (83.3%) patients after the operation. In the dosedependent group 8 (26.7%) patients had cycle regularity before the operation and 21 (70%) had cycle regularity after the operation. This was a significant change in both groups (P<0.001).

**Table 5:** A comparison of cycle regularity, ovulation, and pregnancy between the study groups


Variable		Standard n=30	Dose-dependent n=30	P value

Regularity of cycles				0.22
	Regular	25 (83.3)	21 (70)	
	Irregular	5 (16.7)	9 (30)	
Ovulation				0.11
	Yes	26 (86.7)	21 (70)	
	No	4 (13.3)	9 (30)	
Pregnancy				0.40
	Yes	11 (36.7)	8 (26.7)	
	No	19 (63.3)	22 (73.3)	


Data represented as frequency (%).

We measured progesterone levels in both groups in the first postoperative menstrual cycle. There was no significant difference between the two groups (P=0.11). However, the mean progesterone level in patients with (12.44 ± 2.20) and without ovulation (2.93 ± 0.20) was significantly different (P<0.001). No case of early ovarian failure was observed in the studied subjects.

## Discussion

The results of our study indicated no difference in the number of antral follicles after the operation in both groups. However, the decrease in the number of antral follicles was significant in the standard treatment group. Such results corresponded to those reported by Nasr et al. (26) who observed a meaningful decrease in the number of antral follicles in the ovarian cautery group that had a fixed dose. However, no decrease in the numbers of follicles and the ovarian volume was observed in the ovarian cautery performed with a Harmonic scalpel group. The authors believe that the decrease in the number of antral follicles or the ovarian volume is caused by the adjacent thermal destruction created by the use of electrocautery. The creation of one puncture destroys the ovarian tissue as deep as 4 mL; thus if 4 punctures are made in each ovary, 3.2 mL of the ovarian tissue will be destroyed. The Harmonic scalpel minimizes the amount of ovarian tissue destruction (0.50 mL), which is about 1/8 of the destruction done by the electrocautery. Salem et al. (27) have considered the decrease in the number of follicles as the undesired consequence of LOD. They indicated that the amount of AMH and numbers of antral follicles were reliable indicators of the ovarian reserve. Their measurement in clomiphene-resistant PCOS women without ovulation could be a useful indicator to assess the treatment outcome of LOD. In our study, the dependency of the energy used by the cautery to the ovarian volume might have led to the selection of a more appropriate amount of energy for the ovarian cautery, and therefore caused less damage to the ovarian tissue.

AMH is one of the new predictive indicators of ovarian reserve (28). This hormone can be used as a substitute for determining the age of ovaries because it is related to the number of initial antral follicles, which can reflect the number of residually stored follicles (29). The current study findings indicated a decrease in the AMH, testosterone, and DHEAS levels in both the standard and dose-adjusted groups. The decrease in the amount of AMH in the dose-adjusted group and the decrease in the amount of testosterone in the standard treatment group were statistically significant. These findings did not fully correspond to the results of similar studies. This could be due to the difference in study design, sample size, or genetic and regional differences among the studied patients. Sunj et al. (30) had a vast inclusion criteria that included variables such as weight, acceptable hormonal range, infertility period, etc. in selection of their study population. This could result in decreased generalizability of the achieved results. In their study, only women with an infertility period of fewer than 3 years participated in the research, while the mean infertility period in our study was 4.7 years. Therefore, one of the reasons for the heterogeneity of the results might be the difference in patient selection due to differences in the inclusion criteria. The results of another study on the changes in AMH, testosterone, and free androgen index by unilateral (doseadjusted) and bilateral (fixed dose) ovarian diathermy revealed a significant decrease in AMH, testosterone, and LH levels in both treatment groups. Amer et al. (31) and Elmashad (32) also reported significant decreases in AMH levels following LOD. However, Farzadi et al. (33) reported no such relationship. The meaningful decreases in serum levels of FSH, LH, AMH, testosterone, and free androgen index following LOD were also reported in the study by Salem et al. (27).

Given that the increase in androgens in PCOS is the result of the insulin’s ability to increase the secretion of androgens in ovarian theca cells, the remarkable decrease in the level of androgens after drilling among patients who receive cauterization with a volume dependent dose might be justified by the hypothesized destruction of androgen generating stromal cells. It is believed that the effects of LOD on androgen levels are influenced by the amount of energy entrapped by the ovaries and, for this reason, low doses may be less successful (13).

Both groups had a nonsignficant decrease in DHEAS levels. A review of previous literature has revealed that the existing data on DHEAS are ambiguous. LOD seems to have a minimal effect on adrenal function, even among women affected by hyperinsulinemia, and the improvement of hyperandrogenism is probably secondary to the decrease in LH concentration and reduced androgen production by the ovarian stroma (34).

In our study, the regularity of menstrual cycles increased from 30 to 83.3% in the standard treatment group. The regularity of menstrual cycles in the doseadjusted group increased from 26.7 to 70%. This was a significant increase in both groups. The intra-group changes were different compared to the Zakherah et al. (14) study. In their study, the cycle’s regularity was higher in the dose-adjusted cautery group (87.9%) compared to the fixed-dose cautry group (75.4%); however, similar to our study, its effect on the regulation of the cycles was significant.

In a study by Nasr et al. (26), the occurrence of regular cycles after LOD was similar in both groups (92.8%) and higher than our study results. Takeuchi et al. (35) reported that a regular menstrual pattern was established in 94% of the patients and the rate of oligomenorrhea decreased to 6%. Felemban et al. (16) observed that the occurrence of regular cycles was 80.4% and oligomenorrhea was 19.6% in patients after ovarian cautery. However, Salem et al. (27) reported that among 37 clomiphene-resistant PCOS patients, the cycles regularization was 16.22% three months after the ovarian cautery and 54.06% after six months. Some authors believe that such differences could be due to the different definitions used for the diagnosis of PCOS or the differences in the study populations (26).

In our study, ovulation occurred in 86.7% of patients in the standard treatment group and 70% of those in the dose-adjusted group; 36.7% of patients in the standard treatment group and 26.7% in the dose-adjusted group became pregnant. The findings of our study contradicted those reported by Zakherah et al. (14). In the latter study, the rate of ovulation (81.8 vs. 62.2%) and pregnancy (51.7 vs. 36.8%) in the volume-dependent ovarian cautery group was significantly higher than the fixed thermal dose group. The authors concluded that the adjusted thermal dose on the basis of ovarian volume (60 joules/ cm<sup>3</sup>) in LOD resulted in improved fertility consequences in comparison to the fixed thermal dose (600 joules per ovary) among clomiphene-resistant PCOS patients. The difference between the results of this study and our research might be due to the differences in sample size or racial and regional characteristics. As with our study, the measurement of the ovarian volume was not done in the standard treatment group. Possibly, the ovarian volume of these patients was more or identical to the patients of the dose-adjusted group; therefore, the same intervention might have been done for the patients in both groups.

In a study by Salem et al. (27), 4 (10.81%) pregnancies occurred after three months and 18 (48.65%) after six months, which were less than our study. They mentioned various reasons for the low rate of pregnancy occurrence among their study patients, which included the existence of subtle aetiologies such as hyperprolactinemia, minor anatomical problems, and male reasons such as varicocele. He also mentioned inadequate drilling to induce optimal changes in fertility parameters.

Ramezani et al. (36) examined the cumulative effect of pregnancy after cauterization of polycystic ovaries in clomiphene-resistant patients at Imam Khomeini Hospital in Karaj, Iran, with the following pregnancy rates after surgery: 14.7% (6 months), 36.8% (12 months), 58.8% (18 months), and 76.6% (24 months). However, in this study, the fixed dose method was used for ovarian cauterization in all patients. Although the rate of pregnancy after 6 months (14.7%) was less than the pregnancy rate achieved in our study (26.7% for the dose-adjusted group and 36.7% for the standard group), the rate of pregnancy after 12 months was very close to that of our standard treatment group after 6 months.

Our study had certain limitations; the small sample size which led to a low power, decreased cooperation of patients for the ultrasound study and the postoperative lab tests, as well as the impossibility of performing TVS in all subjects due to limited facilities in this center.

## Conclusion

The results of this study indicated a significant decrease in antral follicles and testosterone in the standard treatment group in comparison to the dose-adjusted group along with a significant decrease in AMH level in the dose-adjusted group. The changes in DHEAS were insignificant in both groups.

Cycle regulation, and the occurrence of ovulation and pregnancy showed that both methods were efficient; however, there were no statistically significant differences. In terms of the effects of ovarian cautery on these variables, neither of the two methods was superior. It is possible that the small number of samples examined and the differences in the sample selection method or the racial and regional differences might have led to the difference in the results of our study with previous researches. Therefore, conducting similar regional studies with a larger sample sizes are highly recommended.
